# Pre-referral rectal artesunate is no “magic bullet” in weak health systems

**DOI:** 10.1186/s12916-023-02777-y

**Published:** 2023-03-30

**Authors:** Manuel W. Hetzel, Jean Okitawutshu, Antoinette Tshefu, Elizabeth Omoluabi, Phyllis Awor, Aita Signorell, Marek Kwiatkowski, Mark J. Lambiris, Theodoor Visser, Justin M. Cohen, Valentina Buj, Christian Burri, Christian Lengeler

**Affiliations:** 1 Swiss Tropical and Public Health Institute, Allschwil, Switzerland; 2 University of Basel, Basel, Switzerland; 3 Kinshasa School of Public Health, Kinshasa, Democratic Republic of the Congo; 4 Akena Associates, Abuja, Nigeria; 5 Makerere University School of Public Health, Kampala, Uganda; 6 Clinton Health Access Initiative, Boston, MA USA; 7 UNICEF, New York, NY USA

**Keywords:** Severe malaria, Rectal artesunate, Artesunate, Case management, Quality of care, Effectiveness, Referral, Antimalarials, Health systems

## Abstract

Severe malaria is a potentially fatal condition that requires urgent treatment. In a clinical trial, a sub-group of children treated with rectal artesunate (RAS) before being referred to a health facility had an increased chance of survival. We recently published in *BMC Medicine* results of the CARAMAL Project that did not find the same protective effect of pre-referral RAS implemented at scale under real-world conditions in three African countries. Instead, CARAMAL identified serious health system shortfalls that impacted the entire continuum of care, constraining the effectiveness of RAS. Correspondence to the article criticized the observational study design and the alleged interpretation and consequences of our findings.

Here, we clarify that we do not dispute the life-saving potential of RAS, and discuss the methodological criticism. We acknowledge the potential for confounding in observational studies. Nevertheless, the totality of CARAMAL evidence is in full support of our conclusion that the conditions under which RAS can be beneficial were not met in our settings, as children often failed to complete referral and post-referral treatment was inadequate.

The criticism did not appear to acknowledge the realities of highly malarious settings documented in detail in the CARAMAL project. Suggesting that trial-demonstrated efficacy is sufficient to warrant large-scale deployment of pre-referral RAS ignores the paramount importance of functioning health systems for its delivery, for completing post-referral treatment, and for achieving complete cure. Presenting RAS as a “magic bullet” distracts from the most urgent priority: fixing health systems so they can provide a functioning continuum of care and save the lives of sick children.

The data underlying our publication is freely accessible on Zenodo.

## Background

**Fig. 1:**
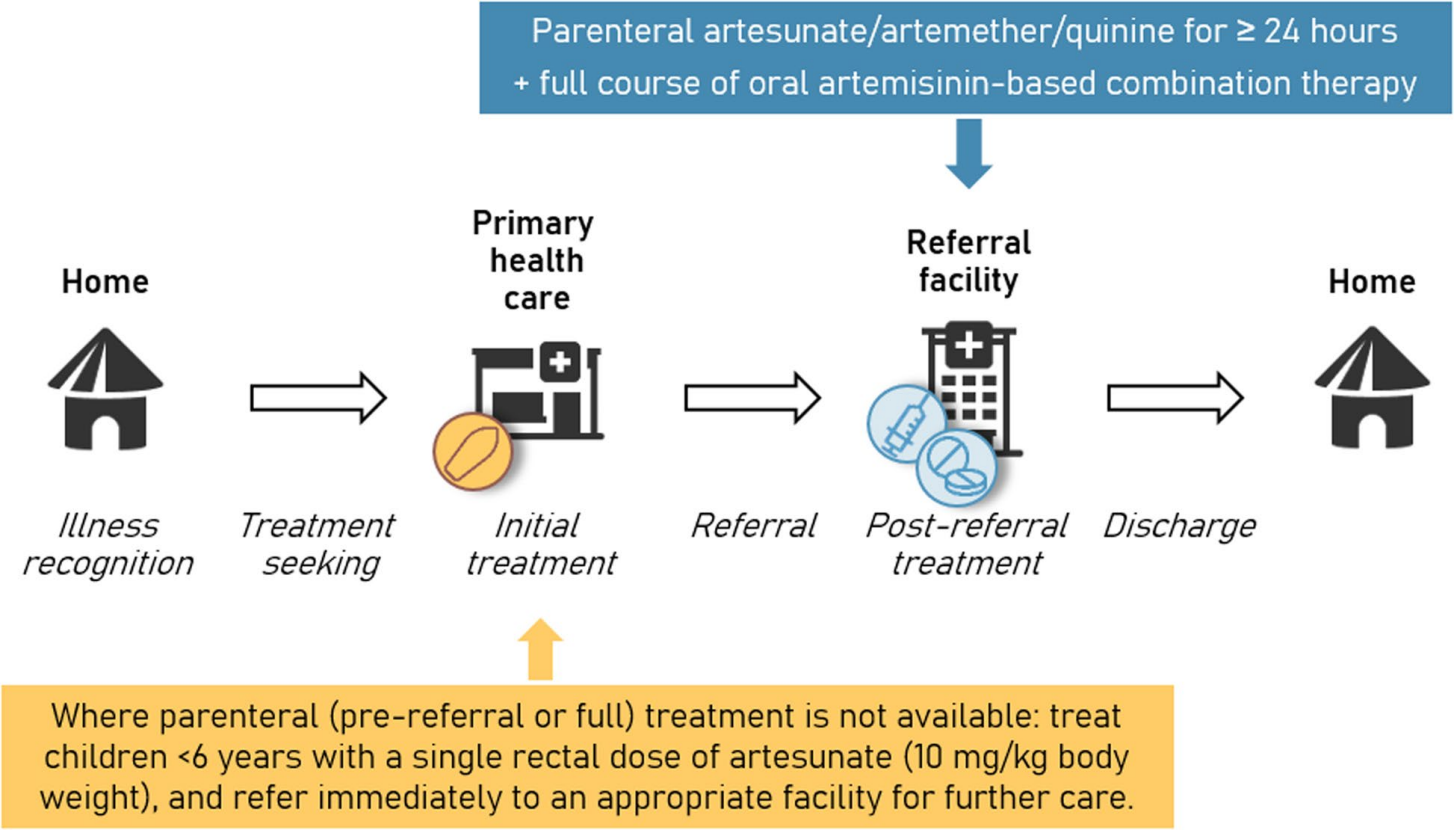
Simplified representation of the continuum of care for severe malaria, required action at each step, and recommended antimalarial treatment at primary and referral levels of care according to World Health Organization (WHO) guidelines [3]

Severe malaria is a potentially fatal or debilitating condition that requires urgent and comprehensive treatment. For a child with severe malaria to be cured, the severity of the illness has to be recognized promptly (both at home and by health care providers), the child needs to be referred to a secondary-level health facility (if not already there), and adequate post-referral case management has to be provided. Unless the entire continuum of care is functional, of adequate quality, and a full package of treatment is administered (Fig. 1), a child is likely to die or suffer from lasting sequelae [1, 2].

**Fig. 2:**
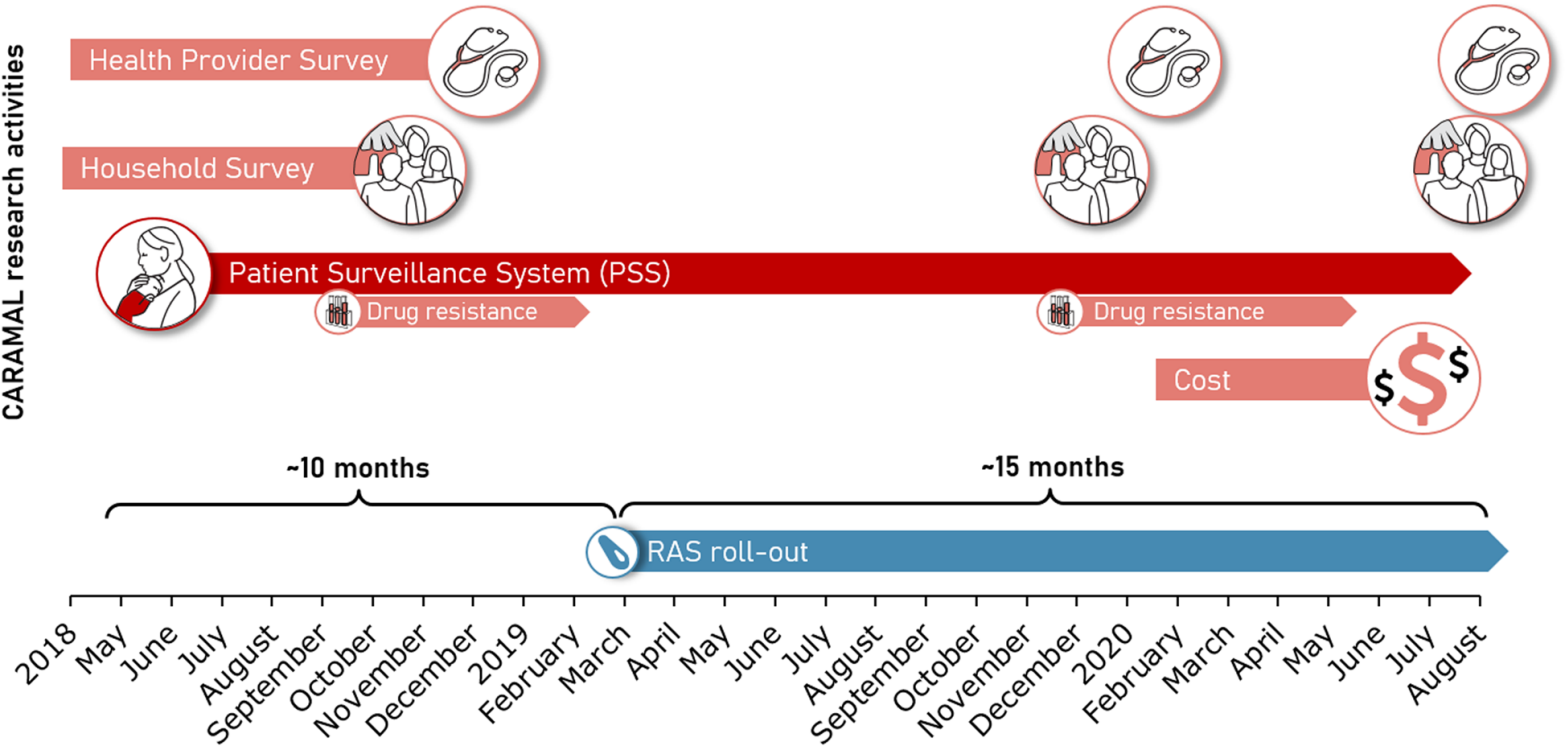
Research activities accompanying the roll-out of pre-referral rectal artesunate (RAS) in the frame of the CARAMAL Project in DRC, Nigeria, and Uganda (modified from [6])

Based on the encouraging results of a randomized controlled trial by Gomes et al. [4], the Community Access to Rectal Artesunate for Malaria (CARAMAL) project was devised to support the roll-out of pre-referral rectal artesunate (RAS) into existing community-based health care services in the Democratic Republic of the Congo (DRC), Nigeria and Uganda, three African countries that jointly contribute 47% of global malaria deaths [5]. CARAMAL aimed to investigate whether and how this efficacious tool could support severe malaria case management in some of the highest malaria burden settings. Hence, the project’s intention was to facilitate the widespread and effective use of pre-referral RAS and contribute to the reduction of severe malaria mortality. A comprehensive description of all components of the CARAMAL project has previously been published, providing essential context to several publications of specific findings of the project’s various research activities (Fig. 2) [6].

CARAMAL was a multi-dimensional implementation research project linked to the scale-up of RAS in real-world settings. It followed the successful pre-qualification of RAS by the World Health Organization (WHO). Both activities were funded by Unitaid in an effort to make pre-referral RAS widely accessible and maximize the life-saving potential of the drug [7, 8].

CARAMAL was implemented between 2018 and the end of 2020. Results were initially made available as pre-prints to facilitate early review by the WHO’s Malaria Policy Advisory Group (MPAG) in October 2021 [9]. Subsequently, a series of peer-reviewed publications was prepared (e.g., [6, 10–15]), including Hetzel et al. in *BMC Medicine* [16]. Additional manuscripts are in preparation and in press (e.g., [17]).

The overall findings of CARAMAL are sobering but clear: the continuum of care for severe febrile illness, including severe malaria, was largely dysfunctional in the study areas, resulting in incorrect and incomplete treatment and unnecessary deaths [6, 16]. We did not conclude from the findings that RAS has no life-saving potential or that the drug might be harmful. However, our inescapable conclusion is that introducing pre-referral treatment with RAS into these weak health care systems is likely to provide little benefit because many children subsequently fail to complete referral [10], and post-referral treatment is often inadequate [15]. The major recommendation emerging from CARAMAL is therefore to urgently address health system constraints along the entire continuum of care, so that fundamental conditions for RAS to be effective are met.

In response to our publication in *BMC Medicine* of health outcomes of children included in the CARAMAL Patient Surveillance System (see Fig. 2) [16], criticism of the study design and of the interpretation of findings was raised [18]. Our critics claim that the impact evaluation was flawed from the start, and heavily criticize the decision of the WHO to qualify the implementation of RAS by adding a requirement to consider health system circumstances for its deployment [19]. This criticism seems to be ignoring the health care realities of high endemicity settings, the wider health system circumstances required for pre-referral RAS treatment to work, and the broad scope and congruent findings of the various CARAMAL project activities [6].

Given the chain of treatment that is required to properly manage a child with severe malaria (Fig. 1), the context in which our health outcome data were generated is of utmost importance. A severely sick child who does not complete referral to an appropriate higher level health facility or who is not treated correctly in these facilities is likely to suffer severe consequences, regardless of whether RAS was administered as a treatment initiation. The original efficacy trial by Gomes et al. [4] already clearly noted this concern: “Referral remains important both to complete the treatment of malaria and to diagnose any other underlying life-threatening infection. […] For referral, appropriate health-care facilities need to be functioning reliably and accessible.” The authors of the commentary interpreted individual calculations out of the important context of evidence presented across several CARAMAL manuscripts, misrepresenting the central findings of the CARAMAL project and some of our conclusions. Several statements in the commentary are factually incorrect and the underlying claims about the supposed negative consequences of our findings (e.g. that our results and analyses — rather than the poor quality of care systematically documented by CARAMAL — are to be blamed for the deaths of children in Africa) are misleading.

The CARAMAL findings and their interpretation are discussed in two recently published viewpoints [20, 21]. Here, we focus on addressing criticism raised in the commentary and correcting major erroneous statements [18].

## Scope and implementation of CARAMAL

CARAMAL was implemented by a consortium which included partners supporting the implementation of pre-referral RAS, and research organizations. All involved partners worked closely with local health authorities and other stakeholders to (i) introduce quality-assured RAS into existing community health care systems, and (ii) gather evidence to inform and support its scale-up across endemic countries. The purported narrow project scope mentioned in the commentary does not reflect the breadth of activities and the overall purpose of the CARAMAL project, as described in detail by Lengeler et al. [6].

The protocol of the CARAMAL project including all of its research activities (Fig. 2) was reviewed by seven Ethics Committees, including the WHO Research Ethics Review Committee. Our analyses were guided by a data analysis plan previously reviewed by WHO. A few additional analyses were performed based on emerging findings, a commonsense practice in observational studies. Hence, we fundamentally disagree with the implicit assertion that any analysis not specified in the original study protocol is therefore invalid. Suggestions that there was no analysis plan, or that there might not have been a study protocol, are simply wrong.

## WHO Information Note and ongoing WHO evidence review

Following the first review of the CARAMAL findings by the WHO MPAG in 2021, WHO issued an Information Note [19]. The content of this document is a non-binding recommendation to countries; it is neither a change of existing policy nor a change of therapeutic guidelines. The Information Note cautioned against the expansion of pre-referral RAS until further guidance is provided by WHO, and recommended that countries using RAS review the conditions under which it is being used currently. The authors of the commentary in *BMC Medicine* have no basis to assert that RAS deployment has been halted as a result, and in reality, CARAMAL project countries and others continue to procure RAS (see, e.g., [22]).

At present, a WHO Evidence Review Group is reviewing the findings of several projects that assessed the implementation of RAS at scale, including CARAMAL. As part of this review, key CARAMAL data are being re-analyzed by independent experts. This comprehensive evidence review is meant to contribute to the development of operational guidance on the safest and most effective use of RAS at scale, a process that has been at the heart of the CARAMAL project from the beginning [6, 23].

It is misleading to claim that the Information Note or the ongoing evidence review is based solely on any particular calculation reported in any one of our manuscripts. WHO and the public have been provided with the breadth of CARAMAL evidence that, taken together, found that the basic conditions under which RAS was previously shown to be effective were not met in our study settings: too many children did not complete referral after receiving RAS, and post-referral treatment was often poor [10, 15]. With no reduced case fatality in children receiving RAS in DRC and Nigeria [16] and an apparent selection of resistant parasites in RAS users in Uganda [17], it is certainly not unreasonable to caution countries that scaling up this intervention requires a robust and resilient health system environment.

## Study design and causality

CARAMAL was not devised to re-evaluate the previously reported efficacy of RAS established in three different study settings [4, 24, 25]. Even though the trial by Gomes et al. [4] had methodological limitations [26, 27], the mechanism through which pre-referral RAS can buy time for a child with severe malaria to reach a higher-level health care facility for comprehensive treatment is plausible. At the same time, the fact that several post hoc sub-group analyses of the trial data had to be performed to identify a group of patients in which RAS was efficacious, and the statistically significant finding of increased mortality in older children receiving RAS, point to the complex mode of action of this treatment in a health system environment. Given this context, we were surprised by the vigor with which the commentary criticizes our analyses in spite of our rather careful interpretation, while showing little concern about the reliability of post hoc analyses in the trial that is often considered the “gold standard” [28]. The suggestion that trial-demonstrated efficacy is sufficient to warrant large-scale deployment of health interventions ignores the paramount importance of a functioning health system for their delivery [29] and persists in promoting “magic bullet” solutions to real-life complexity. Randomized controlled trial (RCT) data is necessary but it is not sufficient. If the efficacy of antimalarial interventions guaranteed effectiveness and population-wide impact, we would not see children dying from malaria today. Cost-effectiveness, coverage of other interventions and their potential interactions, health priorities and available financing are key considerations underlying implementation decisions, as illustrated in discussions about the optimal way of scaling-up the RTS,S/AS01 malaria vaccine [30]. The importance of considering unintended uses of a drug is exemplified by the ban of oral artemisinin monotherapy in spite of its high efficacy [31–33]. The finding that severe malaria is often misdiagnosed further supports a broader health systems strengthening approach over an aggressive push of a single intervention with a narrow indication [34].

We are of course not calling for a ban on RAS. On the contrary, we want RAS to be used in the most effective and efficient way and would have liked nothing better than to see it save lives in our study communities. Unfortunately, CARAMAL found that the narrowly defined scenario under which RAS was shown to work in the trial by Gomes et al. [4] did not exist in the three real-world settings in which we worked. Our highest priority should therefore be to ensure a functioning continuum of care and adequate case management at all levels, which would in turn allow RAS to be utilized to its fullest potential effect. The belief that a “magic bullet” intervention can circumvent the failure of weak health systems is misguided and dangerous. Importantly, it neglects the very “mechanistic reasoning” the authors of the commentary themselves call for [18].

To be very clear: we never suggested a causal link between pre-referral RAS and the observed increase in case fatality over time, neither in Nigeria nor in DRC. On the contrary, we extensively discussed potential non-RAS-related reasons for these time trends and possible confounders beyond what we could adjust for in our study in all three countries [16]. Acknowledging secular trends, we focused on user vs non-user comparisons adjusted as much as possible for changes over time. Nowhere did we claim that RAS itself was directly harmful, thus even a more cautious wording in the abstract, stating that “our study found no evidence that RAS had a beneficial effect” could be intentionally misread.

We acknowledge that cluster-randomized or stepped-wedge randomized trials can generate robust effectiveness estimates because they minimize the issues of confounding and certain other biases. However, in the case of CARAMAL, these study designs were not a feasible option. Our study accompanied the real-world introduction of RAS by local health authorities [5] and it was not up to the research teams to decide who would and who would not receive this intervention. Randomization was also impossible for ethical reasons: under the initial assumption that RAS would have a positive effect, it would have been unacceptable to local authorities to withhold or deliberately delay RAS roll-out. Furthermore, a cluster-randomized trial for the rare fatal outcome would have been of a size that would have made it impossible to carry out in a reasonable time.

The commentary argues that an observational study, particularly a before-after design, cannot prove causality and hence our conclusions are invalid. Yet, even strong proponents of RCTs must realize that situations exist in which an RCT cannot be conducted for ethical or practical reasons, and causality has to be inferred in the absence of an experimental design [35, 36]. The essence of CARAMAL was to understand how RAS would perform as a systemic intervention in the complex real-life situations in which it was expected to be introduced. A randomized study would have defeated this purpose and generated little useful evidence to inform the development of guidelines for large-scale implementation. The broad scope of the CARAMAL project hence required methodological pluralism (i.e., triangulation applying complementary methodologies) much more than it would have benefitted from randomization. Our plausibility approach allowed us to arrive at general conclusions with confidence as they were supported by congruent findings from multiple data sets, multiple methods, and multiple locations [35, 36]. As it turns out, ours was an appropriate choice given that CARAMAL was able to document the major health systems shortfalls that impacted negatively the life-saving potential of pre-referral RAS.

## Analytical issues

The commentary [18] speculates at length on why the reported effect estimates may be biased. We reject outright the suggestion that RAS administration was ascertained poorly. Whether a child received RAS was recorded at enrollment, and corroborated with caregivers. Possible confounding by secular trends in disease severity and treatment seeking, as well as potential confounding by indication have been acknowledged in the article. Importantly, the possibility of a distortion due to COVID-19-related restrictions (implemented in some areas around April 2020) was excluded by an appropriate sensitivity analysis. There is indeed anecdotal evidence (also mentioned in the article) that in Nigeria some CHWs may have given RAS preferentially to sicker children when suppositories were short in supply. The real question, however, is not whether the reported estimates are entirely unconfounded, but whether the confounding can be so severe as to reverse a substantial protective effect of RAS. Drawing on the totality of evidence from CARAMAL referenced above, we can answer this emphatically in the negative. Lastly, we regret that one set of analyses was overadjusted, which resulted in an inflated odds ratio for DRC. The correctly adjusted estimates were reported as well in the same table, and they support our conclusions.

Anyone who wishes to re-examine the calculations underlying our publication in *BMC Medicine* [16] may do so by downloading the dataset which is now freely accessible on Zenodo [37].

## Summary and conclusions

The overall case fatality in our study population was 6.7% in DRC, 11.7% in Nigeria, and less than 1% in Uganda [16]. A total of 154 children (7% in DRC, 19.7% in Nigeria, and 0.4% in Uganda) died *in spite of* receiving pre-referral RAS [16]. Among children directly attending a referral facility and diagnosed with severe malaria, 1.9% died in DRC, 9.6% in Nigeria, and 0.9% in Uganda [6]. All of these children had been in contact with the local health system but the health care services were unable to save their lives. In these settings, children died — and continue to die — because of limited access to comprehensive high-quality care, often on top of difficult socio-economic conditions that promote multi-morbidity.

Drawing on our empirical findings and first-hand experience working in the study sites, we consider our principal finding highly plausible in light of the natural history of severe malaria and the documented functioning of health systems: pre-referral RAS is unlikely to have a beneficial effect on child survival in a context in which children often fail to complete referral and post-referral treatment is inadequate. Presenting RAS as a “magic bullet” in these settings distracts from the most urgent priority: fixing local health systems so they can provide a functioning continuum of care for sick children, from the community to a referral facility.

## Data Availability

The dataset referenced in this article is available in the Zenodo repository: 10.5281/zenodo.5548261 [16, 37].

## Abbreviations

CARAMAL: Community Access to Rectal Artesunate for Malaria project
MPAG: Malaria Policy Advisory Group
RCT: Randomized controlled trial
RAS: Rectal artesunate
WHO: World Health Organization
